# Removal of *Salmonella* Typhimurium Biofilm from Food Contact Surfaces Using *Quercus infectoria* Gall Extract in Combination with a Surfactant

**DOI:** 10.4014/jmb.2101.01014

**Published:** 2021-02-01

**Authors:** Peetitas Damrongsaktrakul, Songsirin Ruengvisesh, Arewan Rahothan, Nuttamon Sukhumrat, Pravate Tuitemwong, Isaratat Phung-on

**Affiliations:** 1 Department of Microbiology, Faculty of Science, King Mongkut’s University of Technology Thonburi (KMUTT), Bangkok 10140, Thailand; 2 Food Safety Center, Institute for Scientific and Technological Research and Services (ISTRS), KMUTT, Bangkok 10140, Thailand; 3 Maintenance Technology Center, ISTRS, KMUTT, Bangkok 10140, Thailand

**Keywords:** *Quercus infectoria* gall extract, *Salmonella* Typhimurium, biofilm removal, food contact surfaces, surfactants

## Abstract

*Quercus infectoria* (nutgall) has been reported to possess antimicrobial activities against a wide range of pathogens. Nevertheless, the biofilm removal effect of nutgall extract has not been widely investigated. In this study, we therefore evaluated the effect of nutgall extract in combination with cetrimonium bromide (CTAB) against preformed biofilm of *Salmonella* Typhimurium on polypropylene (PP) and stainless steel (SS) coupons in comparison with other sanitizers. The minimum inhibitory concentration (MIC) and the minimum bactericidal concentration (MBC) of nutgall extract and surfactants (CTAB and sodium dodecyl sulfate; SDS) were assessed. CTAB showed a more efficient antimicrobial activity than SDS and was selected to use in combination with nutgall extract for removing biofilm. To determine the biofilm removal efficacy, the PP and SS coupons were individually submerged in 2x MBC of nutgall extract (256 mg/ml) + 2x MBC of CTAB (2.5 mg/ml), nutgall extract alone (256 mg/ml), CTAB alone (2.5 mg/ml), distilled water, and 100 ppm sodium hypochlorite for 5, 15, and 30 min. The remaining sessile cells in biofilm were determined. Overall, the greatest biofilm removal efficacy was observed with nutgall extract + CTAB; the biofilm removal efficacy of sanitizers tended to increase with the exposure time. The SEM analysis demonstrated that *S*. Typhimurium biofilm on PP and SS coupons after exposure to nutgall extract + CTAB for 30 min displayed morphological alterations with wrinkles. This study suggests nutgall extract + CTAB may be an alternative to commonly used sanitizers to remove biofilm from food contact surfaces in the food industry and household.

## Introduction

Foodborne diseases are a significant public health concern and also an impediment to social development globally [1]. *Salmonella* spp. have been one of the four key causative agents of foodborne diseases worldwide [2]. According to the U.S. Centers for Disease Control and Prevention (CDC), *Salmonella* spp. cause approximately 1.35 million foodborne infections, 26,500 hospitalizations, and 420 fatalities annually [3]. In Europe, the European Food Safety Authority and European Center for Disease Prevention and Control (EFSA and ECDC) reported 91,662 confirmed human salmonellosis cases in 2017; *S*. Enteritidis and *S*. Typhimurium were the top two serovars associated with the illnesses. The highest risk agent/food pairs were reported to be *Salmonella* in eggs and *Salmonella* in meat and meat products [4]. In Thailand, the Thailand Bureau of Epidemiology, Department of Disease Control, Ministry of Public Health reported 109,491 cases of foodborne illnesses in 2019, with *Salmonella* spp. as one of the major known pathogens causing the diseases [5].

*Salmonella* spp. are facultatively anaerobic, rod-shaped, non-sporeforming, gram-negative bacteria belonging to the family *Enterobacteriaceae* [6]. *Salmonella* spp. can form biofilms, which are complex microbial communities embedded in an extracellular matrix or exopolymeric substances, including polysaccharides, proteins, and nucleic acids [7]. In the food industry, biofilms are undesirable since they serve as a habitat for pathogenic or spoilage microorganisms and can result in cross-contamination from food contact surfaces to food products [8, 9]. Compared to planktonic cells, biofilms are 2 to > 3,000 times more resistant to antimicrobial agents [10]. Therefore, biofilms cannot be effectively inactivated by commonly used sanitizers such as chlorine, peracetic acid, hydrogen peroxide, trisodium phosphate, and quaternary ammonium [9-14]. It has been reported that *Salmonella* spp. can be isolated from food contact surfaces in food processing facilities and domestic kitchens even after cleaning or disinfection [15, 16]. The common food contact surfaces in food processing plants where microbial biofilms exist include filling or packaging equipment, floor drains, walls, cooling pipes, conveyors, collators, racks, hand tools, gloves, and freezers [16, 17]. The presence of *Salmonella* spp. in the food processing areas suggests inefficient cleaning/sanitizing practices, which could increase the contamination level in the food processing facilities, resulting in salmonellosis outbreaks [7].

*Quercus infectoria* (nutgall) is a small tree indigenous to Greece, Asia Minor, Syria and Iran [18]. Upon attack by an insect (*Diplolepis gallae tinctoriae* or *Cynips quercufolii*), galls are formed on young branches on the tree [19]. The galls, also known as nutgalls or oakgalls, are the tree excrescences [20] that contain a mixture of tannin (60-70%), gallic acid, methyl gallate, ellagic acid, and polyphenols. Previous studies have reported that nutgalls possess broad-spectrum anti-inflammatory, antivenom, antidiabetic, antioxidant, and antitumor activities [21]. Also, nutgalls have been shown to possess antimicrobial activities against pathogens (*e.g.* *S. enterica*, *Shigella flexneri*, *Helicobacter pylori*, *Streptococcus mutans*, *Staphylococcus aureus*, *S. pyogenes*, and *Listeria monocytogenes*) [20,22-26]. Many studies have been done to evaluate the antimicrobial activities and pharmaceutical properties of nutgall extracts [18,24,26-28]. However, research on the effect of nutgall extract in removing biofilm from food contact surfaces is limited.

In the food industry, some of the commonly used sanitizers and disinfectants (*e.g.*, peroxide and peroxy acid mixtures, chlorine, carboxylic acids, acid anionic, and iodine compounds) may form toxic by-products (*e.g.*, haloacetic acids, trihalomethanes, and other carcinogenic compounds) [29]. Conventional sanitizers and disinfectants may also contribute to antimicrobial resistance [29-32]. Therefore, the plant-derived extract would be a promising natural alternative for developing sanitizing agents. In this study, the effect of nutgall extract in combination with a surfactant (cetrimonium bromide; CTAB and sodium dodecyl sulfate; SDS) in removing preformed biofilm of *S*. Typhimurium on polypropylene (PP) and stainless steel (SS) coupons was assessed in comparison with other sanitizing agents. The morphological characteristics of biofilm before and after sanitizing treatments were also determined using a scanning electron microscope (SEM).

## Materials and Methods

### Preparation of *Q. infectoria* Gall Crude Extract

Nutgalls were washed with distilled water and then physically crushed using a mortar. The nutgall powder (100 g) was submerged in 95% ethanol (500 ml) at room temperature for seven days. After filtration, the excess solvent was removed using a rotary evaporator (Model R-205, Canada) at 60°C until completely dry. [22].

### Preparation of Surfactant and Nutgall Extract Stock Solutions

The stock solutions of CTAB (Ajax Finechem, Australia) and SDS (Ajax Finechem) were individually prepared by dissolving the surfactants in sterile distilled water to obtain the initial concentrations of 40 and 200 mg/ml, respectively. To prepare the stock solution of nutgall extract, the crude extract was dissolved in 20%dimethylsulfoxide (DMSO; Fisher Scientific, UK) to achieve the initial concentration of 512 mg/ml. The stock solutions were stirred using a magnetic stirrer until completely dissolved prior to use in a microbroth dilution assay.

### Determination of Total Phenolic Content

The total phenolic content of the crude extract of nutgall was determined using a modified Folin-Ciocalteu colorimetric method as described by Dewanto *et al*. [33]. Briefly, 125 μl of the standard solution and 125 μl of the nutgall extract were mixed in separate containers with 0.5 ml of distilled water and 125 μl of Folin-Cioceu reagent. After 6 min, 1.25 ml of 7% sodium carbonate solution was added, followed by addition of water to the final volume of 3 ml. After a 90–min incubation, the samples were measured at 760 nm using a spectrophotometer (BioTek, USA) with the reference blank. The total phenolic content was expressed as milligrams of gallic acid equivalent in a gram of crude extract (mg GAE/g). Gallic acid was used as a standard.

### Preparation of *S*. Typhimurium Culture for Antimicrobial Assay

*S*. Typhimurium ATCC13311 was maintained on slants of tryptic soy agar (TSA; HiMedia, India) at 4°C. Morphology of the culture was confirmed on xylose lysine desoxycholate agar (XLD; HiMedia) and by microscopic observation. Working slants were prepared by transferring a loopful of *S*. Typhimurium to 10 ml of tryptic soy broth (TSB; HiMedia). The culture was incubated at 37°C for 24 h. After a 24-h incubation, the culture was transferred to 10 ml of TSB and incubated for 24 h prior to the assay.

### MIC and MBC Assays

MIC values of the nutgall extract or surfactants were determined using the broth microdilution assay in a 96-well plate. A volume of 100 μl of the extract and surfactants were serially 10-fold diluted in a 96-well plate to obtain desired concentrations. Then, 100 μl of *S*. Typhimurium in 2x TSB was added to the wells to achieve the concentration of approximately 5 log CFU/ml. Negative growth control containing the extract or surfactant in TSB without bacterium was included in the plates. Positive growth control bearing tested bacterium in TSB without antimicrobials was included. The plates were incubated at 37°C for 24 h. After incubation, 30 μl of 0.015%resazurin (Sigma Aldrich Co., USA) was added to the wells and the plate was incubated for 30 min. After incubation, the plate was visually checked. Wells with a red color indicated bacterial growth. The lowest concentrations of the extract and the surfactants without bacterial growth (*i.e.*, no red color) were deemed the MIC [34]. To study the bactericidal activity of the extract and surfactants, 100 μl of the solution from microbial inhibitory wells was spread on TSA plates. Inoculated plates were incubated for 24 h at 37°C. The concentrations of the extract and surfactants resulting in ≥ 3.0 log CFU/ml reduction of tested microorganisms were regarded as bactericidal. The lowest concentrations of the extract and surfactants from three replicates were considered the MBC [35]. Three independent replicates of the study were completed; each replicate was performed in duplicate (N=6).

### Biofilm Formation on Polypropylene and Stainless Steel Coupons

The PP (C.A.P. Intertrade Co., Ltd., Thailand) and SS Type 304 (AEC Industrial Services Co., Ltd., Thailand) coupons (10 × 20 mm) were sanitized in 70% ethanol for 15 min. Then, the coupons were autoclaved at 121°C for 15 min and were dried at 60°C. For biofilm formation, the coupons were transferred to conical tubes containing TSB with approximately 6 log CFU/ml of *S*. Typhimurium. The coupons were partly (1 cm^2^) submerged in the medium, and the remaining parts (1 cm^2^) were exposed to the air-liquid interphase to facilitate coupon removal using sterile forceps. The tubes were incubated at 37°C for 72 h to allow for biofilm formation.

### Biofilm Removal Effect

After a 72-h incubation, the coupons were removed from the conical tubes using sterile forceps and then washed three times in phosphate-buffered saline (PBS; HiMedia) to remove loosely attached cells. The coupons were submerged in conical tubes individually containing 4 ml of 2x MBC of nutgall extract (256 mg/ml) + 2x MBC of CTAB (2.5 mg/ml), nutgall extract alone (256 mg/ml), CTAB alone (2.5 mg/ml), distilled water, and 100 ppm sodium hypochlorite (NaOCl; adjusted to pH 7 using 0.1N HCL) for 5, 15, and 30 min. CTAB and NaOCl solutions were prepared by dissolving CTAB and NaOCl individually in sterile distilled water to obtain the target concentrations. The solution of nutgall extract in combination with CTAB (nutgall extract + CTAB) was prepared by dissolving nutgall crude extract in CTAB solution to achieve the concentration of 256 mg/ml of nutgall extract+ 2.5 mg/ml of CTAB. All sanitizing agents were stirred using a magnetic stirrer. The untreated control samples were included to determine sessile cell numbers of *S*. Typhimurium on coupons without sanitizing treatment.

Following sanitizing treatments, the coupons were removed from the conical tubes using sterile forceps and then washed three times in PBS to remove loosely attached cells or antimicrobial residues. Then, cotton swabs moistened with 0.1% peptone water (PW; HiMedia) were used to scrape the submerged parts (1 cm^2^ × 2 sides) of the coupons. The swabs were transferred to test tubes containing 10 ml of 0.1% PW, followed by vortex agitation for 1 min. The resulting samples were serially diluted in 9 ml of 0.1% PW and then spread on TSA plates. After a 24-h incubation at 37°C, *S*. Typhimurium colonies were enumerated. The limit of detection for plating enumeration was 1.70 log CFU/cm^2^. The experiments were performed in triplicate; two independent samples were tested per replicate (N=6).

### Sample Preparation for SEM Analysis

The PP and SS coupons containing biofilms were treated with sanitizing solutions for 30 min. After that, the coupons were washed with PBS, followed by sterile distilled water. For sample fixation, the coupons were submerged in 4% glutaraldehyde for 2 h at 4°C. After fixation, the coupons were washed with sterile distilled water and then subjected to a 15-min gradual dehydration in 25%, 50%, 75%, 95%, and 100% ethanol, respectively. After drying, the coupon samples were sputter-coated with PdAu and then subjected to SEM (Quanta 450, FEI, USA) observation.

### Statistical Analysis

Populations of *S*. Typhimurium were logarithmically transformed and expressed as means ± standard deviation. Logarithmically-transformed numbers of *S*. Typhimurium were analyzed by two-way analysis of variance (ANOVA) to determine differences (*p* < 0.05) in means, followed by mean separation using Tukey’s Honestly Significant Differences (HSD) procedure. PASW Statistics software version 17.0.2 was utilized for statistical analysis.

## Results

### Total Phenolic Content

The total phenolic contents of ethanolic extract of *Quercus infectoria* gall was 625.82 mg GAE/ g crude extract. The value was in line with the phenolic content (672.13 mg GAE/ g extract) reported in the previous study [36].

### MICs and MBCs of Nutgall Extract and Surfactants

The MIC and MBC of nutgall ethanolic extract against *S*. Typhimurium were 16 mg/ml and 128 mg/ml, respectively. For the surfactants, the MIC and MBC values for CTAB against *S*. Typhimurium were 0.156 mg/ml and 1.25 mg/ml, respectively, while the MIC and MBC values for SDS were > 100 mg/ml.

Several studies have evaluated the antimicrobial effect of *Q. infectoria* gall against pathogenic microorganisms [22,23,25-27,36]. Nanasombat *et al*. [36] reported that the MICs of *Q. infectoria* gall extract against *Bacillus cereus*, *L. monocytogenes*, *Porphyromonas gingivalis*, *S*. Typhimurium, and *Y. enterocolitica* were 0.32, >10, >10, >10, and 0.32 mg/ml, respectively. Their MIC value against *S*. Typhimurium was in agreement with the MIC value (16 mg/ml) of nutgall extract from this study. Voravuthikunchai *et al*. [25] found that the MICs and MBCs of *Q. infectoria* ethanolic extract against *H. pylori* strains ranged from 3.12 to 6.25 mg/ml and 3.12 to 12.5 mg/ml, respectively. Factors affecting MIC and MBC values can include the plant variety [37], the extraction method, and tested microorganisms [21]. In general, gram-positive bacteria are more susceptible to the extract than gram-negative bacteria due to the absence of a lipopolysaccharide (LPS) layer in the outer membrane that serves as an extra barrier to prevent access of bioactive molecules [21]. Although little is known about the mode of action of the nutgall extract, the previous study revealed that the nutgall extract could modify hydrophobic regions of microorganisms and may have partitioned the lipids of the bacterial membrane, resulting in more permeable membranes and, eventually, membrane leakage [26]. The proposed modes of action of the nutgall extract also include the ability to interfere with enzymes, including autolysins and ß-lactamase [23].

In this study, cationic and anionic surfactants were utilized. The cationic surfactant CTAB is a quaternary ammonium compound widely used as a sanitizer and disinfectant for manual processing lines and surfaces in the food industry [38]. SDS is an anionic surfactant generally employed for many cleaning applications and is also highly effective in removing oily stains and residues [39]. CTAB [38, 40] and SDS [35, 39] have been reported to exhibit antimicrobial activities. CTAB could form an electrostatic bond with negatively charged sites on microbial cell walls, leading to stress in the cell wall, cell lysis, and death [40]. It has also been reported that CTAB could induce superoxide stress in microbial cells [38]. SDS has been shown to denature membrane-located proteins and damage microbial cell membranes, resulting in leakage of the cytoplasmic constituents and potentially depolarization of the membrane [35]. In the present study, CTAB was more effective in inhibiting and inactivating planktonic cells of *S*. Typhimurium than SDS; this could be due to the positive charge of CTAB that caused electrostatic attraction with the microbial membranes [35]. Therefore, CTAB was selected to use in combination with nutgall extract for removing *S*. Typhimurium biofilms from PP and SS coupons.

### Biofilm Removal Effect of Sanitizers against Preformed Biofilm on Polypropylene and Stainless Steel Coupons

Tables 1 and 2 present the mean survivors (log CFU/cm^2^) of *S*. Typhimurium sessile cells on PP and SS coupons after treatment with sanitizing agents for 5, 15, and 30 min, respectively. The numbers of sessile cells obtained from untreated PP and SS control samples were 5.66 ± 0.02 and 5.97 ± 0.01 log CFU/ cm^2^, respectively. The greatest biofilm removal efficacy was observed with nutgall + CTAB at 30-min exposure time; the remaining sessile cells embedded in biofilm on PP and SS coupons were 2.14 ± 0.12 log CFU/cm^2^ and 2.20 ± 0.17 log CFU/cm^2^, respectively. Water treatment resulted in a statistical reduction of sessile cells on PP (0.46 to 0.63 log CFU/cm^2^ reduction) and SS (0.73 to 0.87 log CFU/cm^2^ reduction) coupons, suggesting that water may have exerted a rinsing effect on biofilm cells.

PP and SS are food contact surface materials commonly used in the food industry and household [41]. In this study, the effects of nutgall extract + CTAB for removing *S*. Typhimurium biofilms from PP and SS coupons were evaluated in comparison with nutgall extract alone, CTAB, water, NaOCl, and untreated control samples. Overall, the biofilm removal effects of sanitizers against *S*. Typhimurium biofilms on PP and SS coupons followed the trend from greatest to least of nutgall extract + CTAB ≥ NaOCl ≥ CTAB ≥ nutgall extract alone > water. The biofilm removal efficacy of sanitizers seemed to increase with the exposure time. NaOCl is an oxidizing agent widely used for sanitizing food contact surfaces in food processing plants [42]. In the present study, NaOCl was unable to completely remove the biofilms of *S*. Typhimurium at all exposure times. Previous studies also showed that NaOCl failed to completely eradicate microbial biofilms from food contact surfaces [14, 42, 43]. de Souza *et al*. [42] cultivated *S. aureus* biofilms on PP and SS coupons in a meat-based broth at 7 and 28°C. After a 30 s-exposure to NaOCl (250 mg/l), *S. aureus* sessile cells on PP and SS coupons reduced to 1.9-2.4 log CFU/cm^2^ and 2.6-2.7 log CFU/cm^2^, respectively. Corcoran *et al*. [14] reported that 48-h and 168-h *S. enterica* biofilms treated for 48 and 168 h with 500 mg/l of NaOCl (10, 45, and 90-min exposure times) resulted in 0.13-1.1 log CFU/coupon reduction and 0.22-0.97 log CFU/coupon reduction, respectively; a greater reduction of biofilms was observed as the exposure time increased [14]. For CTAB, Wang *et al*. [44] found that 1 mg/ml and 10 mg/ml of CTAB could remove 100% of cells (approximately 5.39 log CFU/cm^2^) of *Salmonella* biofilms at the irreversible attachment phase from SS surfaces. However, Simões *et al*. [40] demonstrated that the application of CTAB singly did not promote detachments of *Pseudomonas fluorescens* biofilms formed on SS slides under turbulent and laminar flow conditions. In this study, nutgall extract + CTAB exhibited the greatest biofilm removal efficacy. This could have been due to the ability of CTAB to disrupt hydrophobic interactions associated with the crosslinking of the biofilm matrix [40], allowing nutgall extract to penetrate the biofilm EPS matrix more efficiently. It is worth noting that this study focuses on the removal of preformed biofilm on food contact surfaces. While evaluation of biofilm formation in the presence of sanitizing agents was not completed in this study, previous work has reported that higher sanitizer concentrations are required to remove preformed biofilms of *S*. Typhimurium than to inhibit biofilm formation on food contact surfaces [32]. To the authors’ knowledge, there has been no research on the biofilm removal effect of nutgall extract in combination with a surfactant. Previous research has demonstrated inhibition of *Streptococcus mutans* biofilm formation in the presence of nutgall extract but did not report the removal of preformed biofilm in the study [24]. It should also be noted that the authors’ study employed the immersion method without additional physical treatment for removing *Salmonella* biofilm from PP and SS coupons. Applying additional physical treatment before or after exposure to nutgall extract + CTAB may therefore have resulted in increased biofilm removal efficacy and a lower concentration of nutgall extract + CTAB required for biofilm removal. In the food processing plants, the commonly used chemical agents can cause antimicrobial resistance in targeted microorganisms [29-32]. Therefore, nutgall extract + CTAB could potentially be utilized as an alternative sanitizer for removing biofilms from food contact surfaces to prevent microbial resistance in food processing plants and households.

### SEM Analysis

**Fig. 1:**
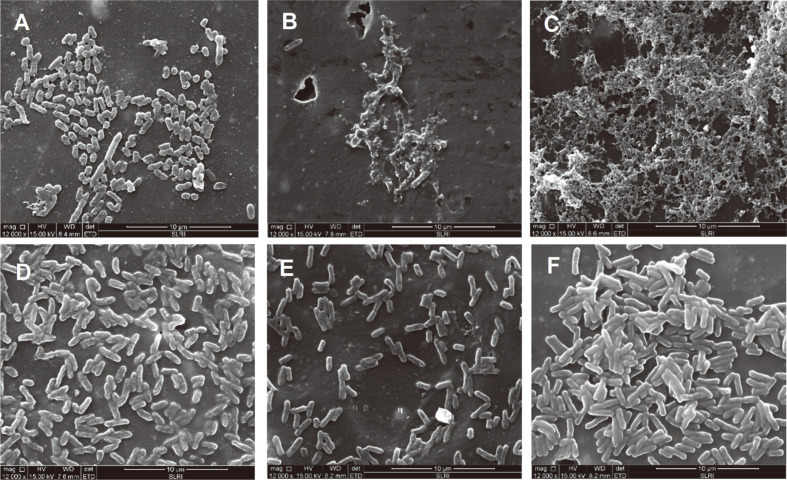
SEM images of S. Typhimurium on polypropylene coupons after treatment with sanitizing agents for 30 min. (**A**) Nutgall extract; (**B**) nutgall extract + CTAB; (**C**) CTAB; (**D**) water; (**E**) NaOCl; (**F**) untreated control. The untreated control sample did not receive sanitizing treatment.

**Fig. 2:**
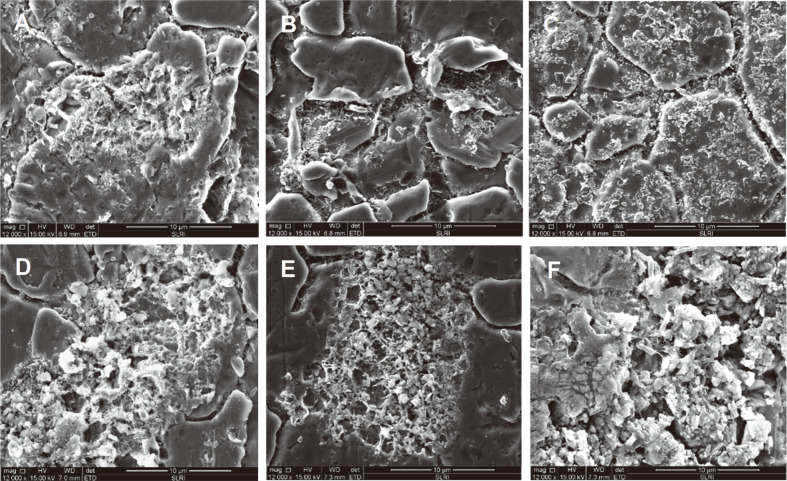
SEM images of *S*. Typhimurium on stainless steel coupons after treatment with sanitizing agents for 30 min. (**A**) Nutgall extract; (**B**) nutgall extract + CTAB; (**C**) CTAB; (**D**) water; (**E**) NaOCl; (**F**) untreated control. The untreated control sample did not receive sanitizing treatment.

The morphology of *S*. Typhimurium biofilms on PP and SS coupons observed under SEM are shown in Figs. 1 and 2, respectively. The untreated control sample on the PP coupon (Fig. 1F) and the sample treated with distilled water (Fig. 1D) exhibited intact biofilm structures with rod-shaped sessile cells. In accordance with the plate count results (Tables 1 and 2), a reduction of sessile cells of *S*. Typhimurium biofilms on PP and SS coupons occurred after treatment with sanitizers. Not all sanitizer treatments resulted in complete removal of *S*. Typimurium biofilms on PP and SS coupons since remaining sessile cells were observed after the treatments. However, the greatest reduction of *S*. Typhimurium sessile cells was observed with the samples treated with nutgall extract + CTAB (Figs. 1B and 2B). A reduction of sessile cells was also observed with samples treated with water (Figs. 1D and 2D), suggesting that water may have displayed a rinsing effect. Treatment with nutgall extract on PP (Fig. 1A) resulted in shrinkage of sessile cells. Biofilm on PP coupons treated with CTAB + nutgall (Fig. 1B) and CTAB (Fig. 1C) showed morphological alterations with wrinkles and ruptures, suggesting that CTAB + nutgall and CTAB alone may have disrupted the cell wall of the sessile cells. The untreated control sample on an SS coupon (Fig. 2F) showed an intact biofilm morphology with dense extracellular polymeric substances (EPS). Excepting exposure to water, biofilm exhibited ruptured EPS after exposure to nutgall extract (Fig. 2A), nutgall extract + CTAB (Fig. 2B), CTAB (Fig. 2C), and NaOCl (Fig. 2E). Previous studies also showed that the application of NaOCl or CTAB to preformed microbial biofilms on food contact surfaces caused morphological changes of biofilm sessile cells but did not result in complete removal of microbial biofilms. Residual cells after sanitizer exposure still remained on the tested surfaces [40, 42, 43]. SEM analysis of microbial biofilms after treatment with plant-derived antimicrobials has been reported in several studies [45-47]. Amaral *et al*. [45] demonstrated that treatment with carvacrol and thymol resulted in a disruption of the typical structure of biofilms and diffuse adherence of *Salmonella* spp. biofilms on polypropylene surfaces. Guo *et al*. [46] showed that exposure to essential oil from *Citrus Changshan-huyou* Y.B. Chang revealed the extensive disruption and architectural changes of *L. monocytogenes* biofilms. Rodrigues *et al*. [47] reported that exposure to oregano and carvacrol essential oil caused morphological alterations with bubbles or spot formations and changes in fimbriae-like filamentous structures on the surfaces of mature *S. aureus* biofilms.

## Conclusions

In this study, overall, the biofilm removal efficacy of the tested sanitizing agents against *S*. Typhimurium biofilms on PP and SS surfaces followed the trend of nutgall extract + CTAB ≥ NaOCl ≥ CTAB ≥ nutgall extract alone > water. The biofilm removal efficacy of the sanitizers seemed to increase with the exposure time. The SEM analysis revealed that *S*. Typhimurium biofilms on PP and SS surfaces after a 30-min exposure to nutgall extract + CTAB exhibited morphological alterations with wrinkles and ruptures. This study indicates that nutgall extract + CTAB may be an alternative to frequently used sanitizers for removing biofilms from food contact surfaces in food processing environments and households.

## Figures and Tables

**Table 1:** Mean survivors (log CFU/cm^2^) of *S*. Typhimurium sessile cells on polypropylene coupons after treatment with sanitizing agents.

Treatment	Mean survivors (log CFU/cm^2^)		

	Sanitizer exposure time (min)		

	5 min	15 min	30 min
Nutgall extract (256 mg/ml)	3.54 ± 0.02^d^	3.14 ± 0.03^f^	2.51 ± 0.07^h^
Nutgall extract (256 mg/ml) + CTAB (2.5 mg/ml)	3.29 ± 0.03^e^	2.84 ± 0.06^g^	2.14 ± 0.12^j^
CTAB (2.5 mg/ml)	3.46 ± 0.06^d^	3.09 ± 0.09^f^	2.35 ± 0.12^i^
Water	5.20 ± 0.03^b^	5.16 ± 0.03^bc^	5.03 ± 0.04^c^
NaOCl (100 ppm)	3.31 ± 0.03^e^	2.94 ± 0.09^g^	2.43 ± 0.10^hi^
Untreated control	5.66 ± 0.02^a^	5.66 ± 0.02^a^	5.66 ± 0.02^a^

Numbers across rows and columns not sharing the same letter are significantly different (*p* < 0.05).

The untreated control represents sessile cells on polypropylene coupons without sanitizing treatment.

**Table 2:** Mean survivors (log CFU/cm^2^) of *S*. Typhimurium sessile cells on stainless steel coupons after treatment with sanitizing agents.

Treatment	Mean survivors (log CFU/cm^2^)		

	Sanitizer exposure time (min)		

	5 min	15 min	30 min
Nutgall extract (256 mg/ml)	3.53 ± 0.02^d^	2.92 ± 0.08^g^	2.61 ± 0.06^h^
Nutgall extract (256 mg/ml) + CTAB (2.5 mg/ml)	3.18 ± 0.05^f^	2.84 ± 0.10^g^	2.20 ± 0.17^j^
CTAB (2.5 mg/ml)	3.57 ± 0.02^d^	3.17 ± 0.04^f^	2.47 ± 0.11^i^
Water	5.24 ± 0.02^b^	5.20 ± 0.02^bc^	5.10 ± 0.02^c^
NaOCl (100 ppm)	3.38 ± 0.04^e^	3.10 ± 0.07^f^	2.60 ± 0.07^hi^
Untreated control	5.97 ± 0.01^a^	5.97 ± 0.01^a^	5.97 ± 0.01^a^

Numbers across rows and columns not sharing the same letter are significantly different (*p* < 0.05).

The untreated control represents sessile cells on stainless steel coupons without sanitizing treatment.
